# Anthropometric Obesity Indices, Body Fat Percentage, and Grip Strength in Young Adults with different Physical Activity Levels

**DOI:** 10.3390/jfmk4030051

**Published:** 2019-07-31

**Authors:** Mustafa Söğüt, Ömer Barış Kaya, Kübra Altunsoy, Cain C. T. Clark, Filipe Manuel Clemente, Ali Ahmet Doğan

**Affiliations:** 1 Faculty of Sport Sciences, Kırıkkale University, Kırıkkale 71450 Turkey; 2 Centre for Sport, Exercise and Life Sciences, Coventry University, Coventry CV1 5FB, UK; 3 Polytechnic Institute of Viana do Castelo, School of Sport and Leisure, 4960-320 Melgaço, Portugal; 4 Instituto de Telecomunicações, 6200-001 Delegação da Covilhã, Portugal

**Keywords:** obesity indices, body composition, grip strength, physical activity

## Abstract

The purposes of this study were to determine whether moderately physically active (MPA) and highly physically active (HPA) male (*n* = 96, age = 22.5 ± 1.7 years) and female (*n* = 85, age = 21.3 ± 1.6 years) young adults differed in their anthropometric obesity indices (AOIs), body fat percentage (BF%), and muscular strength, and also to examine the associations between physical activity level (PAL) and the abovementioned variables. Participants were measured for body height and weight, BF%, waist and hip circumferences, and maximal isometric grip strength. According to their PAL, estimated by the short version of the International Physical Activity Questionnaire, they were assigned to MPA and HPA subgroups. Regardless of gender, results indicated that participants in the MPA groups had significantly higher values of body weight, waist and hip circumference, BF%, and BMI than participants in the HPA groups. No significant differences were found between physical activity groups in terms of grip strength. The AOIs and BF% were found to be significantly and negatively correlated with the PAL in both genders. In conclusion, the findings of the study suggest that high habitual physical activity is associated with lower adiposity markers. However, the differences in the hand grip strength of the contrasting activity groups were negligible.

## 1. Introduction

The consequences of physical inactivity, including a diverse range of chronic and non-communicable diseases is well-documented, such as coronary heart disease [1], breast and colon cancers [2,3], osteoporosis [4], noninsulin-dependent diabetes [5], excess adiposity [6], depression [7], and all-cause mortality [8]. Nevertheless, sedentary lifestyle behaviors are still common worldwide [9], and it presents a major global public health concern.

Accumulating evidence suggests that habitual physical activity has a positive influence on various health outcomes [10,11,12]. The findings of large, recent cohort studies have indicated that physical activity level (PAL) is inversely associated with body fat percentage (BF%), body mass index (BMI), and/or other adiposity indicators [13,14,15,16]. Notwithstanding, inconsistent results have been observed in certain age groups with regard to the relationship between physical activity and muscular strength [17,18,19,20].

Muscular strength is a determinant of physical quality that ensures a lower cardiovascular risk factor [21], a healthy mineral bone density, and high levels of lean mass [22], independently of other fitness variables or sociodemographic measures. Despite there being many ways to assess muscular strength, one of the most common tests carried out in adults is grip strength, which is an important clinical and prognostic value [23]. Usually, strong and positive correlations have been found between hand grip strength and muscle mass [24]. On the other hand, low hand grip strength is associated with increased risk of functional limitations and disability in one’s later years, as well as a general cause of mortality [25]. In spite of such evidence, there is a lack of studies testing the relationship between PALs and strength, and this should be considered to understand the mechanism that explains good maintenance of strength.

Although young adulthood is a critical developmental period characterized by important changes in health status, such as unhealthy weight gain [26,27] and physical inactivity [28], the majority of the relevant examinations have been conducted with children and older adults. Thus, current literature provides limited evidence on the disparities in adiposity and muscular strength in university students with different PALs. Therefore, the purposes of the present study were to examine whether moderately physically active (MPA) and highly physically active (HPA) male and female young adults differed in their anthropometric obesity indices (AOIs), BF%, and muscular strength, and to analyze the relations between PAL and the aforementioned variables.

## 2. Materials and Methods

### 2.1. Participants

A cohort of male (*n* = 96, age = 22.5 ± 1.7 years) and female (*n* = 85, age = 21.3 ± 1.6 years) university students from a state university located in central Anatolia (Turkey) were recruited to take part in the study. They were initially briefed of the measurement procedures and the aims of the study, and then requested to sign informed consent forms. Ethical approval was obtained from the Non-interventional Researches Ethics Board of Kırıkkale University (approval number/identification code of the study is 2019.06.25).

### 2.2. Anthropometric Measurements

Anthropometric assessments were performed in accordance with standardized procedures [29]. Body height was measured with a portable stadiometer (Seca 213, Hamburg, Germany) to the nearest 0.1 cm. Body mass (0.1 kg) and BF% were evaluated by a bioelectrical impedance analyzer (Tanita, BC-418, Japan). Waist and hip circumferences were measured with a flexible steel tape to the nearest centimeter at the smallest circumference between the ribs and the iliac crest and at the level of maximum protuberance of the buttocks, respectively. BMI was calculated by dividing the body weight (kg) by the body height squared (m).

### 2.3. Grip Strength

A digital hand dynamometer (T.K.K.5401 Grip-D, Takei, Japan) was used to assess maximal isometric grip strength. Participants were asked to stay in a standing position and keep one arm straight and parallel to the body. They were requested to hold the dynamometer with their dominant hand and squeeze the handle as hard as possible for three seconds. The highest value acquired from the three trials was used for the analysis.

### 2.4. Physical Activity

The PAL of the participants was estimated via the short form of the International Physical Activity Questionnaire (IPAQ) [30,31]. The IPAQ requires respondents to recall the frequency, duration, and intensity of physical activity they engaged in during the last seven days. Each type of activity (moderate- and vigorous-intensity walking) was then converted to metabolic equivalent (MET) minutes, enabling the determination of total weekly physical activity. Based on their weekly MET minutes, they were divided into two groups—MPA (≥600–3000 MET min/week) and HPA (≥3000 MET min/week) [30,31].

### 2.5. Statistical Analysis

Data were analyzed using SPSS for Windows. Descriptive statistics (mean ± SD) were calculated for all variables. An independent sample t-test was used to ascertain differences between PAL groups. Effect sizes (ES) were quantified to determine the magnitude of differences. Based on the Cohen’s d values, ES were considered as: <0.20 (trivial), 0.20 to 0.59 (small), 0.60 to 1.19 (moderate), 1.20 to 1.99 (large), 2.0 to 3.9 (very large), and >4.0 (extremely large) [32]. A Pearson correlation coefficient was conducted to examine the associations between PAL and other variables. Correlations were categorized as 0.0–0.1 (trivial), 0.1–0.3 (small), 0.3–0.5 (moderate), 0.5–0.7 (large), 0.7–0.9 (very large), and 0.9–1.0 (near perfect) [32].

## 3. Results

Descriptive statistics (mean ± SD), t-test results, and effect sizes for male and female participants are presented in Table 1 and Table 2. Results highlighted that participants in the HPA groups had significantly lower values in BF%, body mass, waist and hip circumference, and BMI than the participants in the MPA groups in both genders. There were no significant differences in age, body height, and grip strength results between groups.

Table 3 presents the correlation coefficients between PAL and other variables for each gender, separately. Results indicated that the PAL, regardless of gender, was found to be significantly and negatively correlated with all AOIs and BF%. On the other hand, no significant associations were found between PAL and grip strength values in both male and female participants.

## 4. Discussion

The aim of the present study was to investigate the possible differences in various adiposity parameters and muscular strength in a group of young male and female adults with distinct physical activity profiles. The results of the study indicated that the participants in the HPA group had significantly lower values in all AOIs and BF% than their MPA counterparts in both genders. Furthermore, regardless of gender, PAL was found to be significantly and negatively correlated with AOIs and BF%. The results, with regard to reported correlation coefficients, are in line with the findings of previous longitudinal [33,34,35] and cross-sectional [36,37] examinations. Concordant with our findings, results of some recent work noted similar observations, that individuals who engaged in greater physical activity were found to have preferential indices of adiposity [13,14].

The results demonstrated that there were no significant differences in grip strength results between activity groups. This observation is in accord with the findings of previous studies conducted with children [38], young adults [39], and older adults [40]. Furthermore, no significant associations were obtained between PAL and grip strength values in both genders. This might conceivably be attributed to comparable body sizes, where trivial and small magnitudes of differences were found between groups for body height and weight, respectively. Furthermore, the participants were grouped by the IPAQ, which does not specifically assess participation in strengthening activities, so may not be sufficiently sensitive. In addition, other possible anthropometric correlates of hand grip strength that were not measured in this study, such as hand size and forearm circumference [41], total arm length and upper arm circumference [42], and hand circumference [43] might contribute to this insignificance, and therefore warrants further examination.

With regard to practical implications, it is conceivable that high PALs and good levels of strength may help to maintain health levels across adulthood. Different studies have suggested that upper and lower body muscular strength are important factors that contribute to a lower risk of mortality in the adult population, regardless of age [44,45]. Although no differences were found between PALs in strength, it is important to highlight that strength is an independent physical quality and should be worked out concurrently with other fitness components (e.g., cardiorespiratory fitness). Thus, a healthy lifestyle should include both cardiorespiratory and muscular strength training/stimuli [46].

## 5. Conclusions

The findings of the present study revealed that higher levels of habitual physical activity is associated with lower adiposity markers in young adults. Nevertheless, differences in hand grip strength of the contrasting activity groups were negligible. It should also be acknowledged that the current study has several limitations. Firstly, the subjective assessment may cause incorrect estimation of physical activity; however, use of the well-validated IPAQ ameliorates some concern. Secondly, in spite of its important advantages (i.e., being relatively inexpensive, portable, and quick), rather than BIA, criterion methods such as underwater weighing or dual-energy x-ray absorptiometry might be used to determine body fat, but nevertheless present significant time and cost restrictions. Nevertheless, it is recommended that future studies expand this observation through using a larger sample and more complex reference methods to measure physical activity levels and body composition.

## Figures and Tables

**Table 1:** Descriptive statistics, t-test results, and effect size values for male participants.

Physical Activity Groups	MPA (*n* = 45)	HPA (*n* = 51)	*t*	*p*	*d*
Age (years)	22.8 (1.8)	22.2 (1.6)	1.861	0.066	0.38
Activity (MET m/w)	1922.1 (799.2)	4883.1 (1203.3)	−14.001	0.001	−2.89
Height (cm)	175.2 (6.3)	175.1 (6.8)	0.082	0.935	0.02
Weight (kg)	71.9 (8.1)	68.4 (7.5)	2.245	0.027	0.46
Waist (cm)	80.1 (5.7)	77.1 (4.9)	2.707	0.008	0.55
Hip (cm)	96.2 (4.8)	93.7 (5.4)	2.347	0.021	0.46
BMI (kg/m^2^)	23.50 (2.7)	22.34 (2.2)	2.285	0.025	0.46
Body fat (%)	13.5 (5.8)	10.5 (3.8)	3.275	0.001	0.67
Grip strength (kg)	47.5 (7.0)	47.9 (6.8)	−0.291	0.772	−0.06

**Table 2:** Descriptive statistics, t-test results, and effect size values for female participants.

Physical Activity Groups	MPA (*n* = 37)	HPA (*n* = 48)	*t*	*p*	*d*
Age (years)	21.2 (1.7)	21.3 (1.6)	−0.324	0.747	−0.07
Activity (MET m/w)	1665.3 (777.5)	4863.2 (821.2)	−18.214	0.001	−3.98
Height (cm)	162.1 (5.6)	161.2 (6.6)	0.705	0.483	0.15
Weight (kg)	60.8 (11.9)	54.8 (8.7)	2.646	0.010	0.58
Waist (cm)	69.9 (8.1)	66.7 (5.2)	2.184	0.032	0.48
Hip (cm)	96.8 (7.8)	93.3 (6.3)	2.257	0.027	0.49
BMI (kg/m^2^)	23.05 (4.2)	21.03 (2.7)	2.703	0.008	0.59
Body fat (%)	27.6 (8.1)	22.4 (6.5)	3.301	0.001	0.72
Grip strength (kg)	27.2 (4.4)	27.3 (3.8)	−0.182	0.856	−0.04

**Table 3:** Correlation results between physical activity level (PAL) and other variables by gender.

Variables	Male		Female	
	*r*	*p*	*r*	*p*
Height	0.012	0.905	−0.016	0.886
Weight	−0.211	0.039	−0.304	0.005
Waist	−0.373	0.001	−0.246	0.023
Hip	−0.284	0.005	−0.296	0.006
BMI	−0.227	0.026	−0.342	0.001
BF%	−0.432	0.001	−0.398	0.001
Grip strength	−0.033	0.746	0.100	0.364
